# Exploring the Addition of Mango Peel in Functional Semolina Sourdough Bread Production for Sustainable Bio-Reuse

**DOI:** 10.3390/antiox13111278

**Published:** 2024-10-23

**Authors:** María Eugenia Chulibert, Pasquale Roppolo, Carla Buzzanca, Antonio Alfonzo, Enrico Viola, Lino Sciurba, Ilenia Tinebra, Angela D’Amico, Vittorio Farina, Daniela Piazzese, Vita Di Stefano, Marcella Barbera, Raimondo Gaglio, Luca Settanni

**Affiliations:** 1 Department of Agricultural, Food and Forest Sciences (SAAF), Università degli Studi di Palermo, Viale delle Scienze, 90128 Palermo, Italy; 2 Department of Biological, Chemical and Pharmaceutical Science and Technology (STEBICEF), University of Palermo, Via Archirafi, 90123 Palermo, Italy; 3 Centre for Sustainability and Ecological Transition, University of Palermo, Piazza Marina, 90133 Palermo, Italy; 4 Department of Earth and Marine Sciences (DiSTeM), University of Palermo, Via Archirafi, 90123 Palermo, Italy

**Keywords:** mango peel powder, functional bread, lactic acid bacteria, sourdough, polyphenols, antioxidant properties

## Abstract

Mango, a tropical fruit celebrated for its delightful fragrance and high nutritional value, generates significant waste during processing, with approximately 35–60% of the fruit being discarded. However, this waste contains valuable components, such as fibre, carotenoids, polyphenols, and other bioactive compounds. In an effort to repurpose mango peel, this study dehydrated it to create mango peel powder (MPP), which was then incorporated into sourdough bread to produce functional breads with enhanced nutritional value. Semolina was replaced with MPP at levels of 5% (MPP-5) and 10% (MPP-10) (*w*/*w*). After dehydration, the mango peel had a yield of 22%, and the procedure used did not cause any organoleptic changes. The bread fermentation process was conducted at 30 °C for 8 h. During dough fermentation, the pH was monitored, showing a value of 4.14 ± 0.02 in the MPP-10 dough. Overall, the MPP-10 bread received a higher score (6.51) than the control (CTR) bread (5.6) and the MPP-5 bread (6.11). The total phenolic content of the fortified breads ranged from 44.760 to 98.931 mg gallic acid equivalents (GAE)/g, and the antiradical activity ranged from 15.213 to 29.461 mmol trolox equivalent antioxidant activity (TEAC)/100 g, depending on the percentage of enrichment.

## 1. Introduction

In the quest to enhance production quality and nutritional value, the food industry is increasingly focusing on sustainable supply chains. Often overlooked, waste materials contain valuable bioactive compounds that can be harnessed to fortify various products [1]. By utilizing by-products from plants and fruits, innovative functional foods have been successfully developed [2]. Mango, a tropical fruit prized for its fragrance, taste, and nutritional value, grows naturally in South Asia and is cultivated in diverse regions worldwide, including India, Mexico, Thailand, and the coasts of Sicily, where favourable weather conditions prevail in particular areas [3].

The steady increase in demand for mangoes in the European market has led to a rise in exports, both within Italy and abroad. Being a climacteric fruit, mangoes undergo rapid ripening after harvest and are often used in the production of fresh products, juices, or dried or freshly cut fruit [4].

A substantial part of the mango (35–60%) is discarded during processing, without adequate treatment, resulting in environmental issues and economic losses [5]. Mango peel is rich in fibre, with approximately 16% to 28% soluble fibre and 29% to 50% insoluble fibre. It also contains valuable phytochemicals, including carotenoids, polyphenols, and other bioactive compounds, which are associated with potential health benefits [6,7,8].

Traditional bread is conventionally leavened using either baker’s yeast or sourdough technology [9]. Sourdough, in particular, benefits from a diverse microbial environment within which lactic acid bacteria (LAB) and yeasts coexist, adding various desirable qualities to the final product [10]. Compared with solely using baker’s yeast, sourdough fermentation offers numerous nutritional and functional advantages. These include lowering the glycaemic index, enhancing the properties of dietary fibre complexes, improving mineral and vitamin absorption, and promoting the production of beneficial metabolites during fermentation, such as peptides and amino acid derivatives like aminobutyric acid [11,12].

In alignment with European initiatives aimed at reducing waste and enhancing the sustainability of the food industry, this study explores the use of mango peel (MP) in powdered form (MPP) as an ingredient for making semolina bread. Although MPP has been used to enrich bread made with wheat flour and baker’s yeast [13,14,15], there are no published studies on incorporating these by-products into sourdough Italian-style breads. The objective of this research is to functionalize traditional sourdough bread produced in southern Italy. By repurposing MPP, valuable polyphenols can be introduced into a staple food within the nutritional pyramid.

## 2. Materials and Methods

### 2.1. Isolation and Identification of LAB from Mango Peel

Mango fruits (*Mangifera indica* L., variety Keitt) were harvested from “Azienda Agricola Tripodo-Collura” in Acquedolci, Sicily, Italy (38°03′27″ N–14°36′38″ E 50 m s.l.m). The peel was manually removed using stainless steel knives and then stored at −20 °C until microbiological analysis.

Presumptive LAB were isolated from the mango peel at the highest cell densities, using the following specific media for plate counting: De Man–Rogosa–Sharpe (MRS), M17, and sourdough bacteria (SDB). Incubation occurred at 30 °C for 48 h anaerobically for MRS and M17 plates, while SDB plates were incubated aerobically under the same conditions. To maximize LAB biodiversity, at least four colonies with similar appearances in terms of shape, size, colour, edge, surface characteristics, and elevation were isolated. All isolates underwent preliminary characterization through Gram staining following the method described by Gregersen [16] and a catalase test as reported by Koneman et al. [17]. Only Gram-positive cultures negative for catalase expression were further purified through successive sub-culturing and stored in glycerol stocks at −80 °C. The acidification kinetics were assessed following the method described by Alfonzo et al. [18], employing a sterile semolina extract (SSE) at 20% concentration as the growth medium. LAB cultures were propagated, washed and inoculated into SSE without maltose. The pH decrease was monitored at 0, 2, 4, 6, 8 and 24 h after inoculation.

The LAB isolates underwent genetic characterization. Overnight cultures were grown in their optimal growth media at 30 °C, and genomic DNAs was extracted from pelleted cells using the InstaGene Matrix kit (Bio-Rad, Hercules, CA, USA) following the manufacturer’s instructions. Crude cell extracts served as templates for PCR. Random amplification of polymorphic DNA (RAPD)-PCR analysis was performed to differentiate the isolates at the strain level. Specifically, the M13 primer, as described by Stenlid et al. [19], was used, and the resulting amplicons were separated and visualized according to Ventimiglia et al. [20]. RAPD profiles were analysed using Gelcompar II software, version 6.5 (Applied-Maths, Sint-Martens-Latem, Belgium). The strains were identified at the species level by 16S rRNA gene sequencing. PCR reactions were performed using the primers fD1 (5′-AGAGTTTGATCCTGGCTCAG-3′)/rD1 (5′-AAGGAGGTGATCCAGCC-3′) as described by Weisburg et al. [21]. The resulting PCR products had a molecular size of about 1600 bp, which was confirmed using agarose gels. After purification with the QIAquick purification kit (Quiagen S.p.a. Milan, Italy), the amplicons were sequenced using the same primers used for PCR amplification at AGRIVET (University of Palermo, Italy). The obtained sequences were then compared with those available in the GenBank/EMBL/DDBJ https://www.ncbi.nlm.nih.gov (accessed on 10 June 2024) and EzTaxon-e databases https://eztaxon-e.ezbiocloud.net (accessed on 10 June 2024).

### 2.2. Mango Peel Powder Production and Characterization

MPP was prepared from mango peels that were first washed under running water (25 ± 1 °C), then sanitised with 2% NaClO solution for 15 min and finally rinsed thoroughly to remove residual chlorine. To ensure uniformity during the dehydration process, the peels were sliced to a thickness of 0.5 ± 0.1 mm and stored at −20 °C until further processing. Initially, the peels underwent treatment in an ultrasonic bath (DU-32, ARGOlab, Modena, Italy) at 30 °C for 15 min at 22 kHz and 70 W, significantly reducing the overall drying time [22]. Subsequently, the mango peels were dehydrated in a tray dryer, as described by Roppolo et al. [23], at 60 °C for 3 h. During the drying process, the trays with peels were weighed every 30 min to halt the process once a 20% dry residue (DR) was reached, thereby preserving bioactive compounds.

The %DR was calculated using the following Equation (1):

%DR = (c − a)/(b − a) × 100(1)

where

a is the weight of the empty tray;
b is the weight of the tray with the product before drying;
c is the weight of the tray with the product after drying.

The colour of raw materials (MPP and semolina) was assessed using a digital colorimeter (Minolta, mod. CR-300; Osaka, Japan) to evaluate the degree of browning based on the CIELab colorimetric system. This system determines colour using three coordinates: L* for brightness (with L* = 0 representing black and L* = 100 representing white), a* for the green/red colour index (where a* = −100 indicates green and a* = +100 indicates red), and b* for the blue/yellow colour index (with b* = −100 signifying blue and b* = +100 signifying yellow). Chroma values (C*), indicating the quantitative attribute of colour intensity, were calculated using Equation, as follows (2):

C* = √(a^2^ + b^2^) (2)

Hue angle (h°), describing the colour tone, was calculated using the following Equation (3):

h° = arctan (b*/a*)(3)

The mango peel was processed using an ultra-centrifugal mill (Fritsch, Pulverisette 14, Lainate, Italy) at a speed of 10,000 rpm for 10 s. The resulting mango peel powder (MPP) was sieved using a Retsch centrifugal apparatus (Mill ZM1, Haan, Germany) equipped with a 250 μm stainless steel ring sieve. The MPP was then analysed for colour, weighed, packed into sealed glass containers, and stored at room temperature (20 ± 1 °C).

MPP underwent microbiological analysis to identify unwanted microbial groups during food fermentation. Ten grams of MPP were homogenized using a BagMixer^®^ 400 stomacher (Interscience, Saint Nom, France) and then serially diluted. All cell suspensions were inoculated in agar media to promote the growth of different microbial groups, as follows: total mesophilic microorganisms (TMM) as well as total psychrophilic microorganisms (TPM) were cultivated on plate count agar (PCA) and incubated aerobically at 30 °C for 72 h and at 7 °C for seven days, respectively; yeasts were cultivated on yeast peptone dextrose (YPD) under aerobic incubation at 28 °C for 48 h; members of the Enterobacteriaceae family were incubated on violet red bile glucose agar (VRGBA) under microaerobic incubation at 37 °C for 24 h; total coliforms were incubated on violet red bile agar (VRBA) under microaerobic incubation at 37 °C for 24 h; pseudomonads were incubated on *Pseudomonas* agar base (PAB) supplemented with cephaloridine sodium fusidate cetrimide under aerobic incubation at 25 °C for 48 h; *Escherichia coli* and *Salmonella* spp. were incubated on Hektoen enteric agar (HEA) under aerobic incubation at 37 °C for 24 h; and *Listeria monocytogenes* was incubated on *Listeria* selective agar base (LSAB) added with SR0140E supplement under aerobic incubation at 37 °C for 48 h. All media and supplements were purchased from Oxoid (Milan, Italy). Plate counts were performed in duplicate.

### 2.3. Sourdough Propagation

A mixture of LAB strains from the Culture Collection of the Agricultural Laboratory, University of Palermo (Italy) was used to initiate a sourdough inoculum. The strains included *Fructilactobacillus sanfranciscensis* SD22, *Weissella cibaria* SD123, *Leuconostoc holzapfelii* SD148, and strains isolated in this study, which were considered intrinsically resistant to the MPP polyphenols. These strains were reactivated from −80 °C glycerol stocks and propagated in SSE at 30 °C for 24 h. The mixed culture was then diluted in sterile tap water to achieve a final volume of 187.5 mL. This cell suspension was added to 312.5 g of semolina, resulting in a 500 g of dough with a dough yield (DY = weight of the dough/weight of semolina × 100) of 160. The cell density in the dough was approximately 10^6^–10^7^ CFU/g. The dough was fermented at 28 °C for 16 h and underwent seven consecutive daily refreshments to develop a mature sourdough inoculum.

The semolina used for bread production was sourced from the brand “La Molisana” (C.da Colle delle Api 100/A, Campobasso, Italy). The labelled nutritional values per 100 g were as follows: 14 g of protein, 70.0 g of carbohydrates, 1 g of fat, 0.3 g of saturated fat, 3 g of fibre, and 0.02 g of salt.

### 2.4. Production of Dough

The bread production process exclusively utilized sourdough developed from the selected LAB strains. No baker’s yeast or salt was added to assess the impact of MPP on LAB performance. The control (CTR) trial (800 g dough) was prepared by adding 228.6 mL of sterile tap water and 457.2 g of semolina to 114.2 g of mature sourdough to reach a final DY = 175. The experimental MPP trials followed the same procedure with the same amount of water and sourdough, but reduced the semolina to 417.1 g and to 377.2 g for the MPP-5 (containing 5% *w*/*w* MPP) and MP-10 (containing 10% *w*/*w* MPP), respectively. The ingredients were mixed using a planetary mixer (model XBM10S Electrolux Professional, SpA, Pordenone, Italy) equipped with a paddle at speed 4 for 15 min. Aliquots of 100 g per dough were transferred into trapezoidal stainless steel baking pans with the dimensions specified by the American Association of Cereal Chemists—Method 10-10B of AACC [24] [143 × 79 mm (top inside), 129 × 64 mm (bottom outside), 57 mm (depth inside)]. The pans were covered with aluminium foil and fermented at 30 °C for 8 h. The baking process included an initial exposure of the doughs to hot air/steam at 200 °C for 5 min, followed by 15 min of convection heat (hot air only) at the same temperature. Each production was performed in duplicate (technical repeats), and the experiment was conducted twice (independent replicates).

### 2.5. Fermentation Monitoring

During sourdough fermentation, the acidification process and the levels of the main fermenting microorganisms were analysed at two time points, as follows: the beginning (T_0_) and the end (T_2_) of fermentation. The pH was measured using a Russell RL060P pH meter (Thermo Fisher Scientific, Beverly, MA, USA). The total titratable acidity (TTA) was determined by titration with 0.1 N NaOH and expressed as the volume of NaOH (in mL) required for 10 g dough. This method follows that of the official American Association of Cereal Chemistry [25].

### 2.6. Microbiological Analysis

Microbial loads were assessed in the raw materials, the doughs immediately after ingredient mixing, and the sourdoughs at different fermentation stages. For each sample, 10 g were suspended in 90 mL Ringer’s solution, homogenized using a stomacher (BagMixer^®^ 400, Interscience, Saint Nom, France) for 2 min at the highest speed, and then subjected to decimal serial dilution. Dilutions were plated and incubated as follows: TMM on plate count agar (PCA), incubated aerobically at 30 °C for 72 h; sourdough LAB on SDB, incubated at 30 °C for 48 h; and total yeasts on yeast extract peptone dextrose (YPD), also incubated at 30 °C for 48 h. To inhibit fungal growth, cycloheximide (10 mg/mL) was added to SDB, while chloramphenicol (0.05 mg/mL) was added to YPD to inhibit bacterial growth. Additionally, the investigation included the detection of members of the Enterobacteriaceae family, total coliforms, and the presence of spore-forming aerobic bacteria, as explained in Section 2.2. Microbiological analysis was conducted in duplicate, and the final results are expressed as log colony forming units (CFU)/g.

### 2.7. Analysis of Bread Attributes

After cooling at room temperature for 30 min, bread quality attributes were assessed as described by Viola et al. [26]. The evaluation included the following parameters: weight loss (%), moisture (%), specific volume (cm^3^/g bread), firmness (N/mm^2^), crumb and crust colour determination, image analysis to determine void fraction (%), cell density, and mean cell area (per cm^2^). The analyses were performed in duplicate.

### 2.8. Total Phenolic Content Analysis of Raw Materials and Bread Samples

Total phenolic content (TPC) was determined using the Folin–Ciocalteu method as described by Viola et al. [26] with slight modifications. Specifically, 0.5 g of each sample (semolina, MPP, CTR, MPP-5, and MPP-10) was added to 8 mL of methanol/water solution (80:20 *v*/*v*). The mixtures were kept in an ultrasonic water bath and sonicated for 45 min. The extracts were filtered through 0.45 μm PTFE filters (Whatman, Milan, Italy). Then, 100 microliters of filtrate solution were added to 625 µL of Folin–Ciocalteu reagent and 120 µL of 7% (*w*/*v*) Na_2_CO_3_ solution. The mixtures were incubated in the dark at 25 °C for 60 min. The reaction formed a blue-coloured complex, with colour intensity proportional to the phenolic compounds present. The resulting colorimetric reaction was measured at 765 nm using a UV–Vis spectrophotometer (UV-1600PC, VWR, Radnor, PA, USA) with methanol as the blank. TPC was calculated by interpolating from a calibration curve of gallic acid (GA), used as the standard (ranging from 0.01 to 0.5 mg/mL). The final results are expressed as milligrams of gallic acid equivalents per gram of the sample [mg gallic acid equivalents (GAE)/g]. Experiments were performed in triplicate.

### 2.9. Antiradical Activity

The antiradical activity of the samples was evaluated using the 2,2-diphenyl-1-picrylhydrazyl (DPPH) and 2,2′-azino-bis (3-ethylbenzothiazoline-6-sulfonic acid) (ABTS) assays, as previously described by Di Stefano et al. [27]. For both tests, scavenging activity was assessed by measuring the decrease in the solution’s colour, which is proportional to the amount of antioxidant in the sample.

One gram of each sample was added to 4 mL of methanol, sonicated for 40 min, and filtered through Whatman 0.45 μm PTFE filters. The filtrate was then subjected to DPPH and ABTS tests. For the DPPH assay, 0.100 mL of the filtrate was mixed with 3 mL of DPPH (60 μM) and incubated in the dark at 25 °C for 30 min. Scavenging activity was measured by spectrophotometric analysis of the absorbance at a wavelength of 517 nm using a UV–Vis spectrophotometer (UV-1600PC, VWR). For the ABTS assay, the ABTS+ radical cation was produced by reacting ABTS stock solution with 2.45 mM potassium persulfate, according to Re et al. [28]. An aliquot of filtered samples (0.100 mL) was mixed with 3 mL of ABTS, and, after 5 min, the absorbance of the mixture was read at 734 nm using a UV–Vis spectrophotometer (UV-1600PC, VWR). Methanol was used as the blank for both assays. Two calibration curves, using Trolox as the standard at increasing concentrations (1–75 μM), were constructed. Experiments were performed in triplicate. The results are reported as mmol Trolox equivalent antioxidant activity (TEAC) per 100 g of sample.

### 2.10. Analysis of Volatile Organic Compounds

Solid-phase microextraction (SPME) was followed by gas chromatography–mass spectrometry (GC–MS) to analyse volatile organic compounds (VOCs) in bread with or without MPP addition. Each sample was chopped, and an amount of 5 g of each sample was exposed to an SPME fibre [30 μm divinylbenzene (DVB)/carbowax (CAR)/polydimethylsiloxane (PDMS)] (Supelco, Bellefonte, PA, USA) at 25 °C for 60 min. The SPME fibre was subjected to 250 °C for 5 min inside the GC injection port after adsorption. A DB-624 capillary column (Agilent Technologies, Santa Clara, CA, USA, 60 m, 60 m, 0.25 mm, 1.40 μm) was used for chromatographic separation. The GC oven temperature ramped up from 40 to 230 °C at a rate of 4 °C/min, remained isothermal for 40 min, and the final temperature was held for 2 min. Mass acquisition was performed in full scan mode in a range from 40 to 400 *m*/*z*, with the interface temperature at 230 °C. VOCs were identified by comparing their mass spectra with the NIST05 library. Results were expressed as percentages, obtained by normalizing peak areas with the total area of significant peaks. Each bread sample was analysed in triplicate.

### 2.11. Sensory Evaluation

Sensory analysis was conducted with a trained panel to evaluate bread attributes. The sensory characteristics of the final bread were analysed according to ISO 6658 guidelines. The judges assessed descriptors related to bread, including crust colour, thickness, crumb colour, porosity, alveolation, and alveolation uniformity [29,30,31]. They also considered sensory aspects, such as bread aroma intensity, unpleasant odour, unpleasant aroma, salty taste, acidic taste, astringent taste, bitter taste, taste persistence, mouth adhesiveness, crispness, and overall assessment. The judges used a visual analogue scale, marking a point between “dislike/low quality” (left end) and “like/high quality” (right end). This scale captured their preferences and acceptability. Results from the hedonic scale were then converted into distances (in centimetres) from the left end of the line. This approach helps quantify sensory preferences and quality perception.

### 2.12. Statistical Analyses

The experimental data were analysed using R, version 3.2.2. Analysis of variance (ANOVA) was used for pair comparisons. Post-hoc analysis to identify differences between groups was performed using Tukey’s test from the Agricolae package, version 1.3-5. Heat map cluster analysis was used to investigate the distribution of VOCs among breads. The sensory traits were graphically represented as a radar chart created with the fmsb package. In all cases, a significance level of 5% was used.

## 3. Results and Discussion

### 3.1. Isolation and Identification of LAB from Mango Peel

No growth was observed on MRS, M17, and SDB media from fresh mango peel dilutions. Consequently, 10 g of mango peel were directly added to 50 mL of MRS and M17 media. Colonies with morphologies consistent with LAB grew on the MRS medium. Phenotypically, the strains were catalase-negative and lacked an external membrane. RAPD–PCR was used to differentiate the strains of presumptive LAB. All isolates sharing a given RAPD profile were considered to represent the same strain. Thus, the dominant LAB populations in the mango peel samples included only four strains. These selected strains underwent 16S rRNA gene sequencing, which revealed the presence of three LAB species. Specifically, three obligate homofermentative LAB were identified as *Latilactobacillus sakei*. This LAB plays a crucial role in fermenting meat products due to its ability to produce lactic acid and enhance food safety and flavour [32]. *Latilactobacillus sakei* has also been used to enhance flavour impact in fermented wheat gluten [33], but its application in bread making remains unexplored.

### 3.2. Characteristics of Mango Peel

The MP yield, calculated from the dry weight of the peels, was 22%, exceeding that previously obtained by De León Monzón [34]. This result highlights the efficiency of our drying method in maximizing yield while effectively preserving the nutritional content of the dried product. Drying is a critical process in food preservation, designed to eliminate moisture from fruits while retaining their nutritional content and sensory qualities. Effective drying techniques are essential for maintaining product quality, including flavour, colour, and texture [35]. Moreover, shorter drying times reduce the risk of prolonged exposure to high temperatures, which can degrade bioactive compounds like anthocyanins, phenols, and vitamins [36]. This preservation process ensures that the nutritional and quality attributes of the food product remain intact. As demonstrated by Roppolo et al. [22], ultrasound treatment reduces the drying time required to achieve a dry residue comparable to that of food dried without its use. Therefore, based on these studies, the same ultrasound-assisted hot-air-drying technology was adopted as a preferred option for the food industry. This approach makes it possible to produce dried products that faithfully retain the characteristics of their fresh counterparts. Both fresh mango peel and powdered mango peel were found to be safe for consumption, as pathogen levels were below the detection limit. In terms of colour, semolina and MPP exhibited significant differences (Table 1).

The semolina sample showed higher brightness (91.57 ± 0.06) compared with MPP (59.13 ± 0.06), indicating that semolina appears much lighter. However, semolina exhibited lower chroma (23 ± 0.14), suggesting a less intense colour. Conversely, MPP showed a higher C* value (35.02 ± 1.51), indicating a more vivid colour intensity, even with a lower hº value (0.80 ± 1.35), corresponding to a light brown hue. These findings demonstrate a trend similar to that observed with avocado powder [26]. The higher brightness of semolina may be linked to its refined nature, whereas the richer chroma and distinct hue of MPP reflect the presence of natural pigments and bioactive compounds in mango peel powder, which are retained during the drying and milling processes. These findings highlight the potential of MPP as a natural colourant and nutrient source, aligning with the trend to incorporate more natural ingredients into food products.

### 3.3. Monitoring of the Fermentation Process

The fermentation process carried out by LAB strains, added as starters, was closely monitored. It began with the preparation of a liquid inoculum in SSE. As the LAB grew in SSE medium, the initial pH decreased from 5.6 to specific values for each strain—3.80 (*F. sanfranciscensis* SD22), 3.88 (*W. cibaria* SD123), 4.38 (*Ln. citreum* SD168) and 3.95 (*Lt. sakei*)—with an average of 4.00 ± 0.26. These findings align with the typical pH changes observed in LAB cultivated in flour and semolina extracts, as documented by Viola et al. [26].

After three days of propagation, a mixture containing all four LAB strains was obtained. This mixture was then used to develop sourdough by adding semolina and sterile tap water. The resulting mature sourdough had a pH of 3.82 and a TTA of 14.88 mL NaOH 0.1 N per 10 g. The pH and TTA values of the sourdough were similar to those recorded in *Ln. holzapfelii* SD148, *Lp. plantarum* SD96, and *Lp. pentosus* SD130 [37]. Additionally, comparable results were found for *Lv. brevis*, *Ln. citreum*, and *W. cibaria* [38] in multiple-species sourdough starter inocula. After seven consecutive days of refreshment, the sourdough was deemed ready for use as a leavening agent in bread production, with a pH of approximate 4, as reported by Corona et al. [39].

The pH and TTA data for doughs at time points T_0_ and T_2_ are presented in Table 2.

Notably, there was a significant negative correlation between pH and TTA (R^2^ = −0.8, *p* < 0.05), consistent with other studies [26,37,40]. Initially, the pH and TTA differed significantly between the control group and both the CTR and MPP-5 doughs (*p* < 0.05). However, by the end of the fermentation process, there were no significant differences in pH and TTA among the groups (*p* > 0.05). The pH values at T_2_ (approximately 4) align with those obtained for powdered-almond-skin-enriched breads [37].

### 3.4. Microbiological Analysis

Table 3 reports the results of dough plate counts, focusing on the primary microorganisms during sourdough fermentation across three bread production trials.

The sourdough, developed using selected LAB, exhibited cell densities of 8.54 log CFU/g for LAB and 7.26 log CFU/g for yeasts. Notably, LAB counts in the doughs (ranging from 7.41 to 7.78 CFU/g) exceeded those of TMM (5.62 to 6.61 log CFU/g), indicating successful transfer of LAB from the sourdough inoculum to the bread doughs. This observation is due to the substantial nutritional requirements of LAB, which were only partially satisfied by PCA [26]. At 0 h post production, yeast levels were significantly lower than those of LAB. Yet, after 8 h of fermentation, LAB cell densities increased for all trials. Notably, LAB levels in both the CTR and MPP-5 doughs were similar (9.20 and 9.14 log CFU/g, respectively), while the MPP-10 dough had a lower density (8.37 log CFU/g). Yeast levels also increased significantly, with no observed differences between doughs (*p* > 0.05). Regarding hygiene indicators, spore-forming bacteria and the Enterobacteriaceae family remained below the detection limit at both T_0_ and T_2_ of fermentation (and are hence not included in Table 3).

### 3.5. Bread Quality Attributes

Baking is a complex process that induces physical, chemical, and biochemical changes in the grain matrix. These changes include water evaporation, volume expansion, the creation of a porous structure, starch gelatinization, protein denaturation, browning, and crust formation [41]. The characteristics of the breads are summarized in Table 4.

No significant differences were observed in weight loss percentage, moisture percentage, and firmness among different breads (*p* < 0.05). As MPP is added, the moisture percentage increases, likely due to its fibre content [42]. However, the weight percentage decreases, as also reported by Pathak et al. [13]. Additionally, the textural properties, such as hardness, cohesiveness, and springiness, also increase with the level of MPP incorporation [13].

The specific volume of the breads decreased as the percentages of MPP increased. Specifically, CTR bread had a volume of 2.45 cm^3^/g, while the MPP-10 bread had a volume of 1.38 cm^3^/g, as also observed by Chen et al. [43]. Image analysis indicated a statistically significant increase in void fraction percentage with the addition of MPP, suggesting that MPP affected the air pockets within the bread structure. Cell density increased with the addition of MPP, consistent with findings from other studies on bread enriched with waste and by-products [26,37]. Interestingly, there was no difference in cell density between MPP-5 and MPP-10, but a significant difference was observed between fortified breads and CTR bread (*p* < 0.05). Alveolation size decreased with MPP addition, and there was a significant difference in alveolation size between CTR bread and both MPP-5 and MPP-10 trials. One of the key factors in consumer decision-making is the visual appeal of a product. Among visual attributes of foods, colour is the most influential character for consumers [4]. Indeed, it is regarded as the most critical element in how consumers perceive quality [44].

The colour of the bread surface is a crucial characteristic closely associated with flavour, texture, and appearance, which are critical factors for consumers. Incorporating MPP changed the colour parameters of both the crust and crumb in the breads. As the percentage of MPP increased, the L* and a* values gradually decreased, while all trials showed negative values for the crumbs. However, despite these results, Chen et al. [43] found that adding MPP increased the L, a, and b* values in bread. Generally, reduced colour variation is preferred as it indicates better preservation of the original colour, which is a crucial indicator of visual quality, and suggests less degradation of pigmented compounds [45].

### 3.6. Total Phenolic Content and Antiradical Activity by DPPH and ABTS Assays

The TPC and radical scavenging activity, measured by DPPH and ABTS assays, were analysed to evaluate the functional attributes of MPP addition. The TPC is fundamental for assessing the presence of phenolic compounds, which are known for their beneficial effects, including antioxidant and anti-inflammatory properties, as well as their ability to protect against oxidative stress [46]. Additionally, the DPPH and ABTS assays allow for the determination of antioxidant capacity. The raw materials results are shown in Table 5.

The results of the TPC analysis in MPP showed a significantly higher value of 276.93 mg GAE/g. Literature data report variable TPC values in mango peels; for instance, Dorta et al. [47] determined TPC values to be around 70 mg GAE/g (dry weight), while Castañeda-Valbuena et al. [48] reported values around 112 mg GAE/g (dry weight). This variability can be attributed to several factors, including differences in mango varieties and preservation methods [47,48]. Interestingly, our findings reveal a notably higher TPC, even in comparison with other fruits [49,50]. Furthermore, previous studies have highlighted how mango peels contain a higher polyphenol content than other parts of the mango, including its pulp [51], indicating the potential of this by-product as a functional additive. MPP also showed substantial antiradical activity, with values of 70.26 mmol TEAC/100 g (DPPH assay) and 281.99 mmol TEAC/100 g (ABTS assay), highlighting the valuable functionality of this by-product.

The TPC values obtained for the control bread (CTR) and mango-peel-powder-fortified bread samples (MPP-5, MPP-10) are reported in Table 6. Bread samples fortified with MPP showed very promising TPC values compared with control bread (CTR). Indeed, our results show that, in experimental bread samples (MPP-5, MPP-10), the addition of MPP increased the TPC, especially in those fortified at 10% (98.93 mg GAE/g). In a study by Pathak et al. [13], bread enriched with 5% MPP exhibited a TPC of 7.57 mg GAE/g. In contrast, our current study found that bread enriched with the same amount of MPP had a significantly higher TPC of 44.76 mg GAE/g, suggesting that experimental bread is a promising carrier for delivering antioxidant compounds. Additionally, in the evaluation of antiradical activity (Table 6), DPPH and ABTS values, expressed as mmol TEAC/100 g, were higher in all fortified bread samples compared with the control, according to percentage of fortification. In particular, bread fortified with 10% of MPP showed values of 29.46 and 116.62 mmol TEAC/100 g for DPPH and ABTS, respectively, compared with the control values of 1.02 and 0.16 mmol TEAC/100 g for DPPH and ABTS, respectively.

Additionally, when compared with bread containing avocado waste powder [26], our study showed higher values for TPC, DPPH, and ABTS. The results from our analyses indicate that the antioxidant potential is improved by enriching bread with different percentages of MPP compared with the CTR bread. Overall, these results underscore the potential of bread fortified with MPP as a valuable vehicle for delivering bioactive compounds.

### 3.7. Volatile Organic Compounds Emitted from Breads Samples

**Figure 1:**
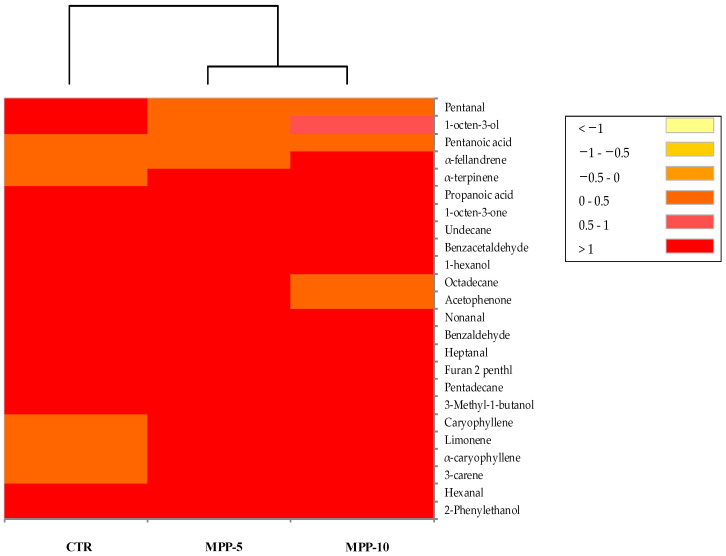
Distribution of volatile organic compounds emitted from breads. The heat map plot depicts the relative concentration of each VOC. Abbreviations: CTR, control bread; MPP-5, experimental bread enriched with 5% (*w*/*w*) of mango peel powder (MPP); MPP-10, experimental bread enriched with 10% (*w*/*w*) of MPP.

VOCs were analysed in bread with and without MPP addition using the SPME–GC/MS technique (Figure 1).

In the control breads, a total of 18 compounds were identified, including four alcohols, six aldehydes, two ketones, and two acids. Short-chain alcohols and fatty acids were linked to sugar fermentation, while higher molecular weight alcohols were found to be associated with amino acid metabolism [52]. Across all breads, the most notable alcohols detected were isoamyl alcohol (3-methyl-1-butanol) and 2-phenylethanol. This observation is common in other studies [38,53,54,55]. Isoamyl alcohol provides balsamic, alcoholic, and malty notes to the bread [52,56]. Interestingly, this compound is commonly found in sourdough fermentation and is produced by various LAB groups, especially lactobacilli, leuconostocs, and weissellas [57]. Phenylethyl alcohol, known for its mild-warm and rose-honey-like odour, arises during fermentation or results from the Maillard reaction [58]. Another significant group in the bread’s aromatic profile consists of carbonyl compounds, with hexanal and benzaldehyde being prominent constituents [53,55]. Hexanal, along with nonanal and pentanal, originates from lipid oxidation [59] and can contribute to a pleasant grassy odour [60]. Benzaldehyde, known for its bitter almond note [61], can derive from the metabolic breakdown or thermal degradation of phenylalanine [62]. Furan compounds arise from thermal reactions such as the Maillard reaction and caramelization [63]. These compounds contribute to the crust’s typical odour notes. Similar VOC profiles have been reported by several authors in bread studies [38,53,54,55].

In bread samples with added MPP, several terpenes were identified. Specifically, monoterpenes such as 3-carene, limonene, α-terpinene, and α-fellandrene, as well as sesquiterpenes such as caryophyllene and α-caryophyllene, were present. Notably, these compounds were absent in the control samples, indicating that they specifically result from the addition of mango. Among these terpenes, 3-carene stood out as the most abundant. Mango is known for its elevated levels of d-3-carene, which typically constitute 60% and 80% of the total terpene content [64,65]. The flavour profiles of the breads were influenced by varying mango fruit percentages, resulting in higher proportions of terpenes and sesquiterpenes in the MPP-10 samples. Terpene compounds are known for their crucial role in enhancing aromatic perception [66]. Therefore, the presence of these compounds in the bread, due to the addition of MPP, indicates that mango significantly influenced the volatile profile of the breads. As previous research on various fruits and vegetables has demonstrated the ability of terpenes to enhance flavour perception and boost consumer preference [67,68,69,70,71], our study suggests that MPP enrichment could positively impact consumer acceptability of the bread [72].

### 3.8. Bread Sensory Attributes

**Figure 2:**
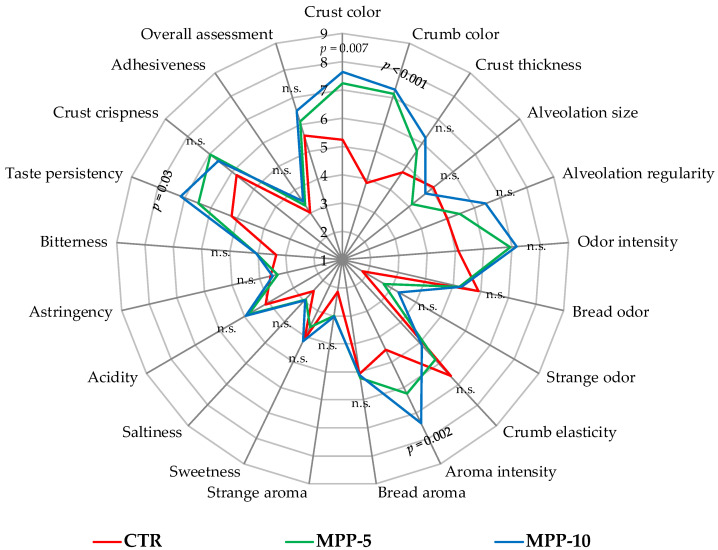
Radar chart of sensory analyses in breads. Statistical differences between doughs are indicated (*p* < 0.05). Abbreviations: CTR, control bread; MPP-5, experimental bread enriched with 5% (*w*/*w*) of mango peel powder (MPP); MPP-10, experimental bread enriched with 10% (*w*/*w*) of MPP; n.s., not significant.

A panel of 13 judges (eight women and five men, aged 23 to 40) conducted sensory analysis on CTR and MPP breads, and the results are graphically presented in Figure 2. Traits significantly different from the CTR bread included crust and crumb colour, aroma intensity, and taste persistency (*p* < 0.05). Except for alveolation size, bread odour, and crumb elasticity, all other traits received higher scores for the MPP breads compared with the CTR bread. In particular, the MPP-10 bread had the highest scores for crust and crumb colour, crust thickness, alveolation regularity, odour and aroma intensity, sweetness, acidity, and taste persistency. However, it is well known that the enrichment of cereal-based fermented products with fruit by-products strongly affects the sensory properties of the final products [26,37]. Overall, the MPP-10 bread received a higher score (6.51) than the CTR bread (5.6) and MPP-5 (6.11). MPP addition undoubtedly influenced sensory characteristics of the breads, with MPP-10 standing out in various sensory aspects.

## 4. Conclusions

This study highlights that MPP, as a by-product, is well suited for functional bread production, enhancing the nutritional profile, particularly in terms of antioxidants. There is a positive correlation between the proportion of added MPP and antioxidant content. The inclusion of 10% MPP in the dough significantly boosts the bread’s antioxidant profile compared with the control bread. Furthermore, sensory evaluations revealed that the aroma and colour of bread made with MPP are highly appreciated. In conclusion, enriched bread holds significant potential as a functional and sustainable staple for consumers.

## Figures and Tables

**Table 1:** Comparative analysis of colour parameters.

Samples	L*	C*	H°	RGB
Semolina	91.57 ± 0.06 ^a^	23 ± 0.14 ^a^	−1.43 ± 0.0 ^a^	
MPP	59.13 ± 1.80 ^b^	35.02 ± 1.51 ^b^	0.80 ± 1.35 ^b^	
*p* value	<0.0001	<0.0001	<0.0001	n.a.

Results indicate mean value ± S.D. (standard deviation) of lightness (L*), chroma (C*), and hue angle (h°) parameters of colour between semolina and mango peel powder (MPP). Data within a column followed by different letters are significantly different (*p* < 0.05). n.a. = not analysed.

**Table 2:** Chemical parameters of doughs.

Time (h)	Parameter	Samples				*p* Value
		Sourdough	CTR	MPP-5	MPP-10	
0	pH	3.82 ± 0.00 ^a^	5.15 ± 0.17 ^b^	4.95 ± 0.00 ^b^	4.58 ± 0.008 ^b^	0.024
	TTA	14.88 ± 3.27 ^a^	3.78 ± 0.78 ^b^	6.23 ± 0.62 ^b^	7.96 ± 2.39 ^b^	0.023
2	pH	n.a.	4.9 ± 0.15	4.69 ± 0.09	4.73 ± 0.13	0.338
	TTA	n.a.	5.97 ± 2.31	7.89 ± 0.15	7.57 ± 1.79	0.550
4	pH	n.a.	4.41 ± 0.22	4.54 ± 0.09	4.50 ± 0.01	0.700
	TTA	n.a.	7.94 ± 1.80	8.80 ± 2.49	8.34 ± 1.73	0.917
6	pH	n.a.	4.16 ± 0.17	4.22 ± 0.22	4.32 ± 0.04	0.955
	TTA	n.a.	8.37 ± 0.38	11.16 ± 1.42	10.85 ± 1.65	0.201
8	pH	n.a.	4.01 ± 0.07	4.03 ± 0.03	4.14 ± 0.02	0.151
	TTA	n.a.	9.25 ± 1.55	11.05 ± 2.26	12.48 ± 1.23	0.316

The results show the mean value ± standard deviation (S.D.) of 4 determinations, performed in 2 technical repeats across 2 independent experiments. Data within a line marked by different letters are significantly different (*p* < 0.05). Abbreviations: TTA, total titratable acidity; CTR, control bread; MPP-5, experimental bread enriched with 5% (*w*/*w*) mango peel powder (MPP); MPP-10, experimental bread enriched with 10% (*w*/*w*) MPP; n.a. = not analysed.

**Table 3:** Microbial loads of doughs.

Microorganisms	Time (h)	Samples				*p* Value
		Sourdough	CTR	MPP-5	MPP-10	
TMM	0	7.6 ± 0.54 ^a^	6.20 ± 0.14 ^b^	6.61 ± 0.41 ^b^	5.62 ± 0.44 ^b^	0.00987
	8	n.a.	7.23 ± 0.44	7.11 ± 0.12	6.77 ± 0.08	0.391
Sourdough LAB	0	8.54 ± 0.93	7.52 ± 1.31	7.41 ± 0.24	7.78 ± 0.69	0.316
	8	n.a.	9.20 ± 0.17 ^a^	9.14 ± 0.11 ^a^	8.37 ± 0.25 ^b^	0.026
Yeasts	0	7.26 ± 0.14 ^a^	6.33 ± 0.00 ^b^	6.19 ± 0.14 ^b^	5.96 ± 0.32 ^b^	0.00826
	8	n.a.	7.37 ± 0.09	6.89 ± 0.27	7.28 ± 0.03	0.247

Results indicate mean value ± S.D. (standard deviation) of 4 plate counts (carried out in 2 technical repeats for 2 independent experiments), expressed as log CFU/g. Data within a line followed by different letters are significantly different according to Tukey’s test. Abbreviations: TMM, total mesophilic microorganisms; LAB, lactic acid bacteria; CTR, control bread; MPP-5, experimental bread enriched with 5% (*w*/*w*) of mango peel powder (MPP); MPP-10, experimental bread enriched with 10% (*w*/*w*) of MPP; n.a. = not analysed.

**Table 4:** Quality attributes of bread samples.

Attributes	Samples			*p* Value
	CTR	MPP-5	MPP-10	
Weight loss (%)	14.87 ± 1.35	14.55 ± 5.15	12.98 ± 2.59	0.116
Humidity (%)	46.11 ± 0.98	47.77 ± 3.75	55.21 ± 4.39	0.138
Specific volume (cm^3^/g)	2.45 ± 0.66 ^a^	1.97 ± 0.15 ^ab^	1.38 ± 0.47 ^b^	0.01
Firmness (N/mm^2^)	0.034 ± 0.006 ^a^	0.036 ± 0.006 ^b^	0.047 ± 0.004 ^c^	<0.0001
Void fraction (%)	40.39 ± 2.32 ^a^	46.57 ± 1.29 ^b^	50.94 ± 1.80 ^c^	<0.0001
Cell density (n/cm^2^)	60.44 ± 1.31 ^a^	90.0 ± 2.83 ^b^	96. ± 0.40 ^b^	0.02
Mean cell area (cm^2^)	0.72 ± 0.02 ^a^	0.54 ± 0.02 ^b^	0.47 ± 0.04 ^b^	0.005
Crust colour				
Brightness	56.67 ± 9.0 ^a^	51.39 ± 7.3 ^b^	50.92 ± 4.9 ^b^	0.006
Readness	8.24 ± 4.61	8.66 ± 7.18	6.99 ± 2.82	0.461
Yellowness	34.13 ± 3.34	32.87 ± 11.74	30.26 ± 2.16	0.135
Crumb colour				
Brightness	68.02 ± 2.12 ^a^	57.86 ± 3.96 ^b^	54.52 ± 2.74 ^c^	<0.0001
Redness	−3.36 ± 0.19 ^a^	−1.01 ± 0.63 ^b^	−0.32 ± 0.32 ^c^	<0.0001
Yellowness	22.01 ± 0.60 ^a^	19.69 ± 0.93 ^b^	22.39 ± 0.94 ^ac^	<0.0001

The results show the mean value ± standard deviation (S.D.) of 4 determinations, performed in 2 technical repeats across 2 independent experiments. Data within a line marked by different letters are significantly different (*p* < 0.05). Abbreviations: TTA, total titratable acidity; CTR, control bread; MPP-5, experimental bread enriched with 5% (*w*/*w*) mango peel powder (MPP); MPP-10, experimental bread enriched with 10% (*w*/*w*) MPP; n.a. = not analysed.

**Table 5:** Antioxidant and antiradical activity of semolina and mango peel powder.

Samples	TPC (mg GAE/g DM)	DPPH (mmol TEAC/100 g DM)	ABTS (mmol TEAC/100 g DM)
Semolina	5.02 ^a^	0.24 ^a^	0.44 ^a^
MPP	276.93 ^b^	70.26 ^b^	281.99 ^b^
SEM	35.25	9.05	0.07
*p* value	0.002	<0.0001	0.0043

Results indicate mean values of 6 determinations (carried out in triplicate for 2 independent productions). Abbreviations: TPC, total phenolic content; mango peel powder (MPP); SEM, standard error of the mean. Letters that are different within the same column indicate significant difference (*p* < 0.05).

**Table 6:** Antioxidant and antiradical activity of control and fortified bread samples.

Samples	TPC (mg GAE/g DM)	DPPH (mmol TEAC/100 g DM)	ABTS (mmol TEAC/100 g DM)
CTR	5.21 ^a^	1.02 ^a^	0.16 ^a^
MPP-5	44.76 ^b^	15.21 ^b^	75.37 ^b^
MPP-10	98.93 ^c^	29.46 ^c^	116.62 ^c^
SEM	6.13	2.55	10.52
*p* value	<0.0001	<0.0001	<0.0001

Results indicate mean values of 6 determinations (carried out in triplicate for 2 independent productions). Abbreviations: TPC, total phenolic content; CTR, control bread; MPP-5 bread, experimental bread enriched with 5% (*w*/*w*) of mango peel powder (MPP); MPP-10 bread, experimental bread enriched with 10% (*w*/*w*) of MPP; SEM, standard error of the mean. Letters that are different within the same column indicate significant difference (*p* < 0.05).

## Data Availability

All data included in this study are available upon request by contacting the corresponding author.
